# Cosmic Silicate Surfaces Catalizing Prebiotic Reactions: Atomistic Modeling on the Polymerization of HCN

**DOI:** 10.1021/acsearthspacechem.5c00166

**Published:** 2025-11-08

**Authors:** Niccolò Bancone, Stefano Pantaleone, Gerard Pareras, Piero Ugliengo, Albert Rimola, Marta Corno

**Affiliations:** † Departament de Química, 16719 Universitat Autònoma de Barcelona, 08193 Bellaterra, Catalonia, Spain; ‡ Dipartimento di Chimica and Nanostructured Interfaces and Surfaces (NIS) Centre, 9314 Università degli Studi di Torino, via P. Giuria 7, 10125 Torino, Italy

**Keywords:** cosmochemistry, periodic surface modeling, heterogeneous catalysis, diaminomaleonitrile, comets

## Abstract

Hydrogen cyanide, HCN, is a fundamental building block in astro- and cosmochemical environments, known for its ability to form prebiotically relevant molecules such as nucleobases. Although its polymerization is inhibited under the cold, dilute conditions of the interstellar medium, the higher temperatures of more evolved rocky bodies, combined with the presence of mineral surfaces, can catalyze the reaction. In this study, we use atomistic simulations grounded on the density functional theory (DFT) to elucidate the complete tetramerization pathway of HCN to diaminomaleonitrile (DAMN) and diaminofumaronitrile (DAFN), catalyzed by the crystalline Mg_2_SiO_4_ forsterite (120) surface. Results demonstrate that the intrinsic acid–base properties of the surface facilitate chemical bond formation/cleavage needed for HCN oligomerization, lowering activation barriers by ∼120–220 kJ mol^–1^ with respect to the gas-phase. Kinetic analyses reveal that the reactions are feasible at temperatures above 300 K, particularly under conditions present in warm, rocky bodies such as asteroids, meteorites, and planetary surfaces. The presence of water further accelerates key steps by assisting proton transfer processes. These findings support a model in which Mg-rich silicate minerals (abundant in the early Solar System) may have directly catalyzed the formation of complex organic molecules, which, in turn, are precursors of more complex biomolecules, thereby contributing to the essential chemical inventory for the emergence of life on early Earth and other primitive planets with propitious conditions.

## Graphical abstract

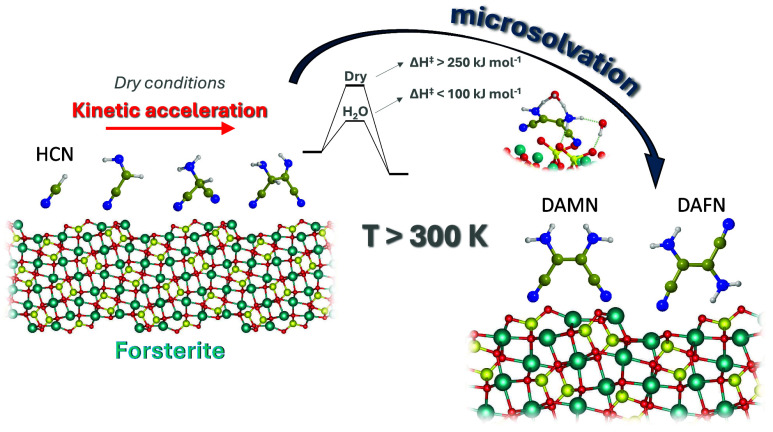

## Introduction

Hydrogen cyanide (HCN) is a ubiquitous molecule detected in various astronomical environments, spanning from the more pristine interstellar clouds, presolar bodies like comets and carbonaceous chondrites, to rocky protoplanetary atmospheres and primitive planets.^1–6^ It possesses an enhanced ability to polymerize and form a diverse array of products, some with prebiotic potential, including adenine.^7–9^ Oligomeric species, possibly deriving from HCN, have been identified in evolved astrophysical environments, as evidenced by the detection of nucleobases within the organic fractions of the Murchison and Orgueil meteorites.^10–12^ Moreover, possible HCN-derived polymers have been observed in Comet Halley^13^ and could also be present in Saturn’s satellite Titan and other evolved bodies.^14,15^ Further evidence of nucleobases synthesized in space was obtained in the asteroids Ryugu and Bennu, sampled for the first time in the recent missions Hayabusa2 and OSIRIS-REx, respectively.^16,17^ These findings suggest that prebiotic compounds could have been introduced to the primordial Earth via exogenous delivery, and, once there, they could have further evolved to form crucial species involved in the origin of life.^18^

Despite its high reactivity and the diversity of its potential products in space, the gas-phase conversion of HCN into more chemically complex compounds in the interstellar medium is significantly hindered by the low temperatures, with rate-limiting steps characterized by potential energy barriers as high as 200–300 kJ mol^–1^,^19^ thereby preventing its polymerization.^20–22^ Base-induced catalysis in solution, instead, facilitates the reaction by promoting the deprotonation of HCN, giving rise to nucleophilic CN^–^ anions, which in turn can react with neutral H_*x*_C_*x*_N_*x*_ species, and enhancing polymerization of pure HCN.^7,23–25^ Similarly, solid water surfaces can favor such mechanism by helping proton transfers from adsorbed HCN to the ice surface,^26^ while radical initiation on defective Si–O^·^ silica surfaces can successfully lead to the formation of 1,3,5-triazine from pure HCN.^27^

Ionic Mg oxides and silicates, abundant minerals in astronomical rocky bodies, are promising catalysts for the prebiotic polymerization of HCN due to the bivalent properties of their surfaces, constituted by both exposed O^2–^ Lewis bases and Mg^2+^ Lewis acids.^28^ Infrared (IR) measurements identified an increasing reactivity of HCN at 300 K when catalyzed by (from lowest to highest) crystalline Mg_2_SiO_4_, amorphous Mg_2_SiO_4_, and MgO,^29^ a trend consistent with an increasing basicity of their surface-exposed O^2–^ anions. High-resolution mass spectrometry analysis of the samples reported the presence of different products with *m*/*z* = 80–177, including small fractions of adenine on all materials. On both the crystalline and amorphous Mg_2_SiO_4_ samples, the most abundant identified product was diaminomaleonitrile (DAMN), a thermodynamically stable HCN tetramer form, in agreement with what was previously reported for basic reaction mixtures.^30,31^ The lightest detected HCN oligomer was aminomalononitrile (AMN, the HCN trimer), while the iminoacetonitrile (IAN, the HCN dimer) was not detected, as it is a short-lived intermediate.

Such results show evidence of the possibility of catalytic processes taking place in the rocky fraction of comets and chondritic bodies, where Mg silicates appear both in their amorphous and crystalline phases and represent the majority of the mineral fraction.^32–34^ For these reasons, in previous works by some of us, we explored the properties of crystalline Mg_2_SiO_4_ forsterite surfaces and the adsorption of gaseous HCN on them, in which efficient deprotonation and strong perturbations of the HCN bonds upon adsorption were observed, already at low temperatures.^35,36^ More recently, as a follow-up work, the results on the catalytic properties of forsterite surfaces of different stabilities—(120), (101), and (111), with surface energies of 1.52, 1.78, and 1.99 J m^–2^, respectively, at the PBE-D*N level of theory^35^—toward the dimerization of two adsorbed HCN molecules to form IAN were reported.^37^ The catalytic effect of forsterite was attributed to two main factors (similarly to what was observed in the liquid phase^23,24^ ): (i) the activation of HCN through its deprotonation by surface-exposed O^2–^ Lewis basic atoms and (ii) the stabilization of transition states for reactions occurring on surface-exposed Mg^2+^ Lewis acidic metal centers. Energy barriers computed on forsterite surfaces were between 149 and 269 kJ mol^–1^ smaller than those in the gas-phase, varying depending on the specific facet. Moreover, the direct formation of IAN, involving a C–C bond, was favored on the surface, at variance with the gas-phase, where the isomer imidoformyl isocyanide (with a C–N bond instead) is formed first. Remarkably, low-energy barriers were predicted on the stable and extended (120) surface (in detriment to less stable ones) since this surface represents an optimal trade-off between the acidity/basicity of the surface-exposed Mg^2+^/O^2–^ pairs,^37^ resulting in high reaction rates and a ready production of IAN through a concerted mechanism. Moreover, we found HCN-adsorption complexes on the (120) surface to well reproduce experimental observations,^36^ further supporting their adoption as reactants for the HCN self-reactivity.

In the present work, we aim to expand the HCN/forsterite reactive systems toward the prebiotic oligomerization of HCN. The focus of the study is directed toward the catalytic effect of the (120) forsterite surface under different temperature conditions, with particular attention to its influence on the reaction barriers. Remarkably, silicates like forsterite, ubiquitously present as the main constituent of asteroids and comet interiors, are always in contact with water, either in the amorphous icy form or as thin liquid films wrapping the mineral core.^38–40^ The liquid water results from heating processes such as body collisions or radioactive decay of ^40^K. It is, therefore, important to study the role of tiny amounts of water on the polymerization paths and how they may affect the kinetics of the process. Accordingly, the effect of the presence of water on the efficiency of the investigated reactions has also been considered. The cosmochemical implications of our findings from an experimental and observational perspective are discussed.

## Computational Methods

All atomistic simulations were performed with the CP2K code (v. 9.1.0),^41^ which mixes plane waves and Gaussian basis sets. The PBEsol functional^42^ was adopted. Valence electrons were described with a double-ζ valence-polarized MOLOPT basis set and a plane-wave energy cutoff of 600 Ry,^41,43^ while core electrons were described through Goedecker–Teter–Hutter pseudopotentials.^44^ Dispersive intermolecular interactions, misdescribed in plain PBEsol, were introduced through the Grimme’s D3 empirical term, including three-body interactions and the Becke–Johnson (BJ) damping function, which avoids near singularities at short distances.^45–50^ All the stationary points of the characterized potential energy surfaces (PESs), namely, reactants, products, intermediates, and transition states, were optimized at this level of theory.

The periodic Mg_2_SiO_4_ forsterite (120) surface slab model, recovered from previous works by some of us,^35,51^ was used here and is depicted in Figure 1. The shorter *a* parameter was doubled to avoid intercell interactions between the adsorbed molecules, and an empty space of around 25 Å was left between slab replicas along the nonperiodic *z* direction. The isolated slab structure was fully optimized (both atomic positions and the 2D periodic cell parameters) at the current PBEsol-D3­(BJ) level of theory, resulting in the following parameters: *a* = 12.043, *b* = 13.884, *c* = 40.000 Å, and α = β = γ = 90.0°.

**Figure 1:**
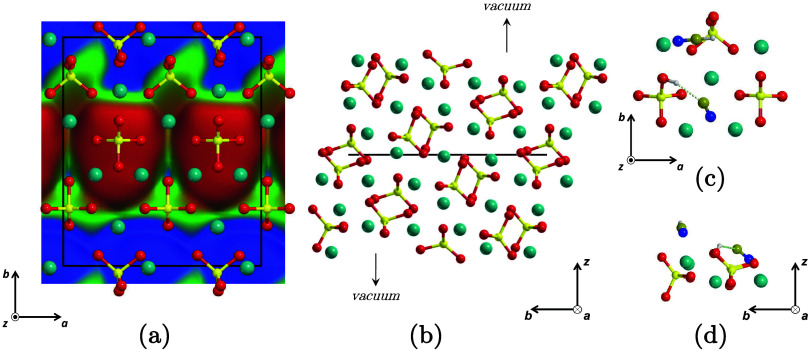
(a) Top view of the Mg_2_SiO_4_ forsterite (120) periodic surface model superimposed on the electrostatic potential map (red = negative; green = neutral; blue = positive). For the sake of clarity, only the most exposed atoms are shown. (b) Side view of the same surface model, showing all atoms. (c, d) Top and side zoom-in of two adsorbed HCN molecules, one of which is deprotonated by an exposed silicate. For axes perpendicular to the reading plane, the × symbol indicates an in-going axis and the • symbol represents an out-going one. Color coding: dark cyan, Mg atoms; red, O atoms; yellow, Si atoms; blue, N atoms; brown, C atoms; white, H atoms; black, unit cell.

The minima along the PES (i.e., reactants, products, and intermediates) were localized with the Broyden–Fletcher–Goldfarb–Shanno (BFGS) algorithm,^52–55^ adopting the default convergence criteria for positions and gradients. At each optimization step, the convergence of the SCF energy was set to 10^–7^ Hartree. The minima nature of each structure was confirmed by vibrational harmonic frequency calculations using the finite difference method implemented in the CP2K code. To minimize the computational cost, only the atoms belonging to the adsorbates, e.g., H, C, N, and, when present, H and O atoms of water, were considered. Localization of the transition states (TSs) was carried out by adopting the climbing image-nudged elastic band (CI-NEB) algorithm.^56^ TS optimizations were performed by relying on the dimer method in CP2K,^41^ with convergence threshold values tightened to one-third of those adopted in minima optimizations, relying on the conjugate gradient (CG) algorithm.^57,58^ The convergence threshold of the SCF energy was set to 10^–9^ Hartree to better describe the PES curvature around the saddle point. Frequency calculations confirmed the real nature of the TSs, for which one (and only one) imaginary frequency associated with the reaction coordinate was identified.

To improve the accuracy of the energetics of the reactions, onto the PBEsol-D3­(BJ)-optimized stationary points, single-point energy calculations at BHLYP-D3­(BJ), coupled with the Ahlrichs’ VTZP basis set for HCN and a smaller contracted Pople basis set including polarization functions for forsterite atoms,^59–62^ were carried out with the CRYSTAL23 periodic code.^63^ The same correction at the BHLYP-D3­(BJ) level was applied in a previous study by some of us on the HCN dimerization on forsterite surfaces.^37^ In the present work, we characterized the accuracy of this scheme against DLPNO-CCSD­(T) in computing energy barriers and reaction energies for the rate-determining steps of the HCN tetramerization, namely, CN^–^ + H_*x*_C_*x*_N_*x*_ nucleophilic attacks and intramolecular proton transfers, when catalyzed by a small forsterite nanocluster^64^ (see Table S1). It shows that computed BHLYP-D3­(BJ) energy barriers are very similar to the DLPNO-CCSD­(T) ones, with deviations no greater than 4.3 kJ mol^–1^, while deviations in the order of 5–28 kJ mol^–1^ are obtained in reaction energies, confirming that the adopted level of theory is particularly suitable for tracing kinetic insights in this reactive system. Relative Gibbs energies at 300 K (Δ*G*(300)) were computed by applying thermal corrections to the BHLYP-D3­(BJ)//PBEsol-D3­(BJ) potential energies.

Unimolecular kinetic rate constants for the simulated reactions were calculated by adopting the Rice–Ramsperger–Kassel–Marcus (RRKM) transition state theory,^65–68^ which was adapted for surface reactions in a freely available code developed by some of us.^69^

## Results and Discussion

As previously mentioned in the Introduction, the oligomerization of HCN proceeds by successive additions of HCN to the organic H_*x*_C_*x*_N_*x*_ structure. Figure 2 shows the elementary steps of the reaction: first, the reactivity between two HCN molecules leads to IAN (HCN dimer), the second addition (HCN + IAN) leads to AMN, and the last one to aminoiminosuccinonitrile (AISN), a tetramer species. From AISN, two possible processes can take place: (i) direct tautomerization, involving a set of proton transfers yielding DAMN, or (ii) the rotation around the main C–C bond followed by tautomerization via proton transfer, yielding diaminofumaronitrile (DAFN).

**Figure 2:**
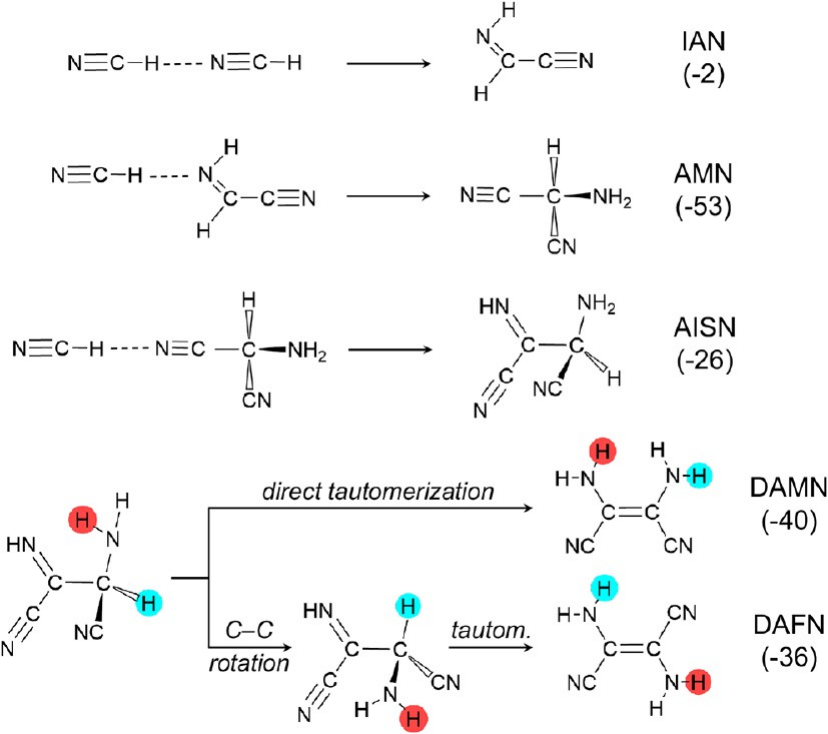
Elementary steps of the HCN tetramerization through successive HCN additions forming the dimer iminoacetonitrile (IAN), the trimer aminomalononitrile (AMN), and the tetramer aminoiminosuccinonitrile (AISN). AISN can yield diaminomaleonitrile (DAMN) or diaminofumaronitrile (DAFN). For each elementary step, the BHLYP-D3­(BJ)//PBEsol-D3­(BJ) gas-phase Gibbs reaction energy at 300 K (Δ*G*(300)) is reported in parentheses, in kJ mol^–1^.

The mechanistic details of these reactions in the gas-phase are not presented here, as they are extensively described in ref 19, but the Δ*G*(300) values of these processes calculated here are shown in Figure 2 for the sake of comparison when occurring on the surfaces, since the specific adsorbate/site interactions may affect the thermodynamics.

### HCN Dimerization: IAN Formation

The Δ*G*(300) profile for the dimerization of two HCN molecules to form IAN on the Mg_2_SiO_4_ forsterite (120) surface is shown in Figure 3.^37^ The adsorption of HCN occurs with the N-end of the molecule coordinating one or two surface-exposed Mg^2+^ centers. The adsorption of two HCN molecules as reactant species is considered here (structure **1** of Figure 3). The reaction is initiated by the deprotonation of one HCN by a surface-exposed O^2–^ atom (TS_1_^dept^, Δ*G* ^‡^(300) = 35 kJ mol^–1^), forming structure **2**, which exhibits a stable SiOH^+^···CN^–^ H-bonded ion pair. The reaction proceeds with the nucleophilic attack of CN^–^ on the C atom of the second (molecularly adsorbed) HCN, followed by the spontaneous protonation of the available N-end of the resulting product (TS_1_^CC^, Δ*G* ^‡^(300) = 48 kJ mol^–1^), forming finally IAN (structure **3**). While the overall process is exothermic (Δ*G*(300) = −21 kJ mol^–1^), intermediate **2** is more stable (Δ*G*(300) = −45 kJ mol^–1^) than the IAN in **3**, preferring the formation of **2**. Nevertheless, we cannot exclude a priori that other possible surface···adsorbate conformations, not investigated here, could instead favor IAN or, complementarily, that the deprotonation energy associated with the formation of **2** could be channeled in the subsequent reaction steps. Indeed, as mentioned in the introduction, IAN is a short-lived intermediate that is not observed experimentally in reacting mixtures of HCN, suggesting that it can, once formed, rapidly evolve to further products. This reactive HCN + HCN → IAN path is the same as that reported in ref 37, but adopting a different quantum chemical methodology. Despite this, the results are in full agreement.

**Figure 3:**
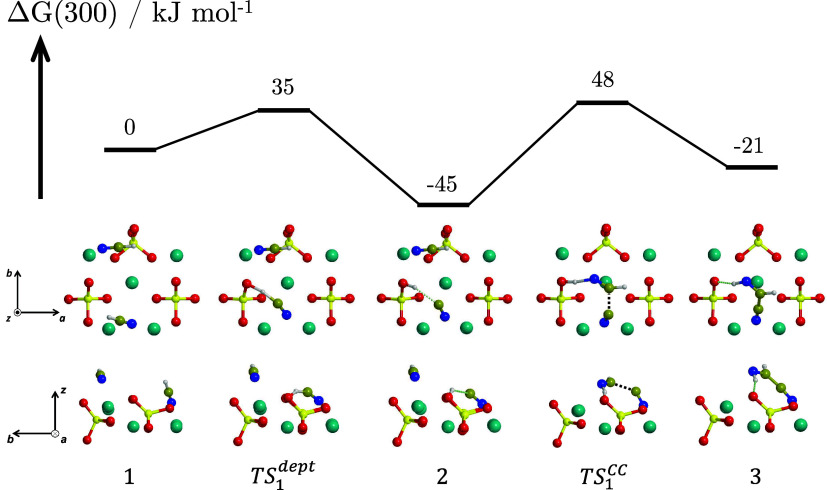
BHLYP-D3­(BJ)//PBEsol-D3­(BJ) Δ*G*(300) profile, in kJ mol^–1^, for the formation of the HCN dimer (IAN) from two HCN molecules adsorbed on the Mg_2_SiO_4_ forsterite (120) surface. Both the top and lateral views of the structures are represented. For the sake of clarity, only the most exposed surface atoms are shown. Color coding: dark cyan, Mg atoms; red, O atoms; yellow, Si atoms; blue, N atoms; brown, C atoms; white, H atoms.

Similar mechanisms for the catalyzed HCN dimerization are reported in water environments,^7,23–25^ where the reactants are activated by basic species, while acidic species stabilize TSs and products. On forsterite, these roles are played by the surface-exposed O^2–^ and Mg^2+^ atoms, respectively.^37^ According to the mechanism proposed by Jung and Choe,^19^ instead, the dimerization step in the gas-phase involves a N–C coupling between two neutral HCN, followed by isomerization, the rate-determining step showing an intrinsic potential barrier of 312 kJ mol^–1^ (CBS-QB3//B3LYP/6-31G­(d) level of theory). Moreover, the reaction energy on the surface (Δ*G*(300) = −21 kJ mol^–1^) clearly indicates a higher exothermicity with respect to the gas-phase as computed here (Δ*G*(300) = −2 kJ mol^–1^, see Figure 2), consistent with the presence of stabilizing IAN/forsterite interactions.

### HCN Trimerization: AMN Formation

HCN oligmerization proceeds with the addition of another HCN molecule to the IAN dimer.^7,25^ The Δ*G*(300) profile for this process is shown in Figure 4.

**Figure 4:**
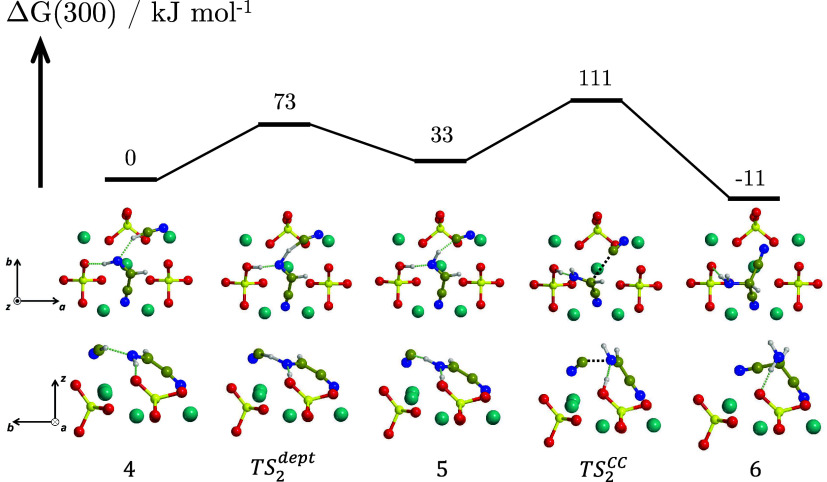
BHLYP-D3­(BJ)//PBEsol-D3­(BJ) Δ*G*(300) profile, in kJ mol^–1^, for the formation of the HCN trimer (AMN) from one HCN molecule and IAN adsorbed on the Mg_2_SiO_4_ forsterite (120) surface. Both the top and lateral views of the structures are represented. For the sake of clarity, only the most exposed surface atoms are shown. Color coding: dark cyan, Mg atoms; red, O atoms; yellow, Si atoms; blue, N atoms; brown, C atoms; white, H atoms.

The newly added HCN is stabilized by an H-bond with IAN and by Mg^2+^ coordination (structure **4** of Figure 4). The reaction proceeds in a similar way to IAN formation, but in this case, HCN donates the proton to the = NH group of IAN, which, in turn, deprotonates toward the surface (TS_2_^dept^, Δ*G* ^‡^(300) = 73 kJ mol^–1^), forming structure **5** as an intermediate. Compared to the previous stage, this deprotonation step is thermodynamically less favored and presents a higher energy barrier, probably due to a poorer stabilization of the resulting CN^–^: in structure **2** (Figure 3), it interacts directly with the surface-exposed Mg^2+^ and the SiOH^+^ counterion, while in structure **5** (Figure 4), it interacts directly with the neutral *E*- isomer of IAN.

The local kinetic barrier at 300 K for the C–C nucleophilic attack (TS_2_^CC^, Δ*G* ^‡^(300) = 111 kJ mol^–1^) amounts to 78 kJ mol^–1^, 15 kJ mol^–1^ smaller than the one for IAN formation (93 kJ mol^–1^) and appreciably smaller than the potential energy barrier associated with the rate-determining step in the gas-phase (202 kJ mol^–1^).^19^ Similar to IAN, AMN is mildly more stable than the corresponding reactants (Δ*G*(300) = −11 kJ mol^–1^), confirming the exothermicity of trimerization.^7,19,23,26^ However, the formation of AMN on the surface is less favorable than in the gas-phase (Δ*G*(300) = −53 kJ mol^–1^, see Figure 2), at variance with IAN formation. This is because, at the geometry of **6**, out of the four possible anchoring points of AMN (the two nitrile groups and the two H atoms of the amine group), only one nitrile and one H atom interact relevantly with the surface, hence making the process less favorable.

Sandström et al.^25^ reported a reaction free energy at 278 K of −59 kJ mol^–1^ for the production of AMN in a HCN solution (using as a solvent the polarized continuum model). This value is comparable to the one computed here in the gas-phase, pointing to the robustness of the reaction energies in the gas-phase and in solution, similarly to further reactions of DAMN.^70^ This is because reactants and products undergo comparable stabilization under isotropic solvation, in contrast to the reaction on the solid forsterite surface.

### HCN Tetramerization: DAMN and DAFN Formation

#### AISN Tetramer Formation

To form the tetramer, a new HCN molecule needs to be added to the AMN. Adopting the mechanism of the previous steps, HCN deprotonates and carries out a nucleophilic attack on AMN. Nevertheless, a favorable AMNH^+^/CN^–^ configuration is not stable in this specific adsorption site and, accordingly, a different mechanism has been considered, represented in Figure 5. In this new mechanism, the HCN → HNC isomerization takes place first, aided by a surface-exposed SiO group assisting the proton transfer from the C-end to the N-end (TS_1_^iso^, Δ*G* ^‡^(300) = 37 kJ mol^–1^).

**Figure 5:**
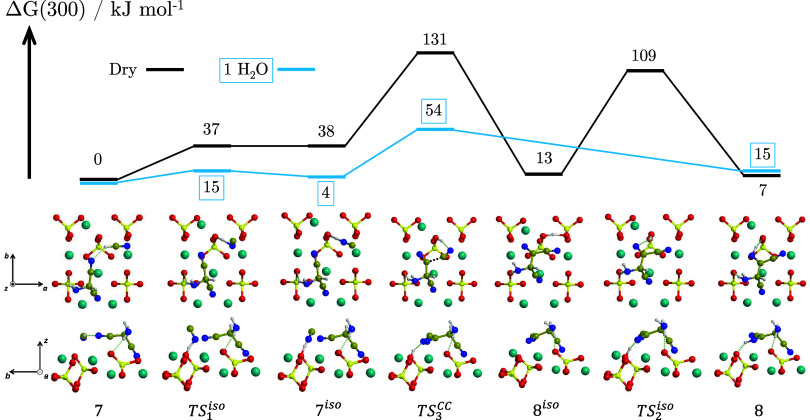
BHLYP-D3­(BJ)//PBEsol-D3­(BJ) Δ*G*(300) profile, in kJ mol^–1^, for the formation of AISN from one HCN molecule and AMN adsorbed on the Mg_2_SiO_4_ forsterite (120) surface in dry conditions (‘Dry’, black) and in the presence of one water molecule assisting proton transfers (‘1 H_2_O’, blue). Both the top and lateral views of the dry structures are represented. Relevant structures in the presence ofone water molecule are shown in Figure 6. For the sake of clarity, only the most exposed surface atoms are shown. Color coding: dark cyan, Mg atoms; red, O atoms; yellow, Si atoms; blue, N atoms; brown, C atoms; white, H atoms.

Upon introduction of zero-point energy, thermal and entropic corrections, the newly formed intermediate (structure **7** ^iso^) lies 1 kJ mol^–1^ higher in energy than TS_1_^iso^. This occurs because (i) the actual potential energy difference between TS_1_^iso^ and structure **7** ^iso^ is small (they are 42 and 41 kJ mol^–1^, respectively) and (ii) the transition frequency associated with the bond formation/breaking within the process produces a nonfavorable zero-point energy difference toward structure **7** ^iso^. The nucleophilic C–C attack now occurs between the HNC and AMN (TS_3_^CC^, Δ*G* ^‡^(300) = 131 kJ mol^–1^), concerted with a proton transfer from HNC to a surface-exposed SiO group. The resulting intermediate (structure **8** ^iso^) is the deprotonated form of AISN. To finally form AISN (structure **8**), the surface proton is transferred to the nearest N atom (TS_2_^iso^, Δ*G* ^‡^(300) = 109 kJ mol^–1^). In the corresponding gas-phase mechanism, the rate-determining step is the HCN + AMN addition (N–C bond formation), showing an intrinsic potential energy barrier of 269 kJ mol^–1^ (CBS-QB3//B3LYP/6-31G­(d)),^19^ thus confirming the strong catalytic effect exerted by the surface.

On the opposite, the highest energy barrier steps for the AISN formation on the surface involve proton transfers (TS_1_^iso^ and TS_2_^iso^). Such processes can be aided by the presence of water molecules adopting a proton relay mechanism, in which a water molecule assists the proton transfers by simultaneously accepting and donating a proton, thus reducing the energy barriers. To this end, one water molecule was introduced to act as a proton transfer assistant for HCN deprotonation and a second one close to the nitrile group exposed to the nucleophilic CN^–^ attack, facilitating the concerted reprotonation of the product (see TS_1_^iso^ and TS_3_^CC^ in Figure 6). While the total amount of water molecules is two, each transfer is assisted by only one water molecule, for which we refer to this system as ‘1 H_2_O’, as opposite to the ‘Dry’ system. The presence of small quantities of water molecules is a reasonable scenario considering the relatively high abundance of water on cosmic refractory materials.

**Figure 6:**
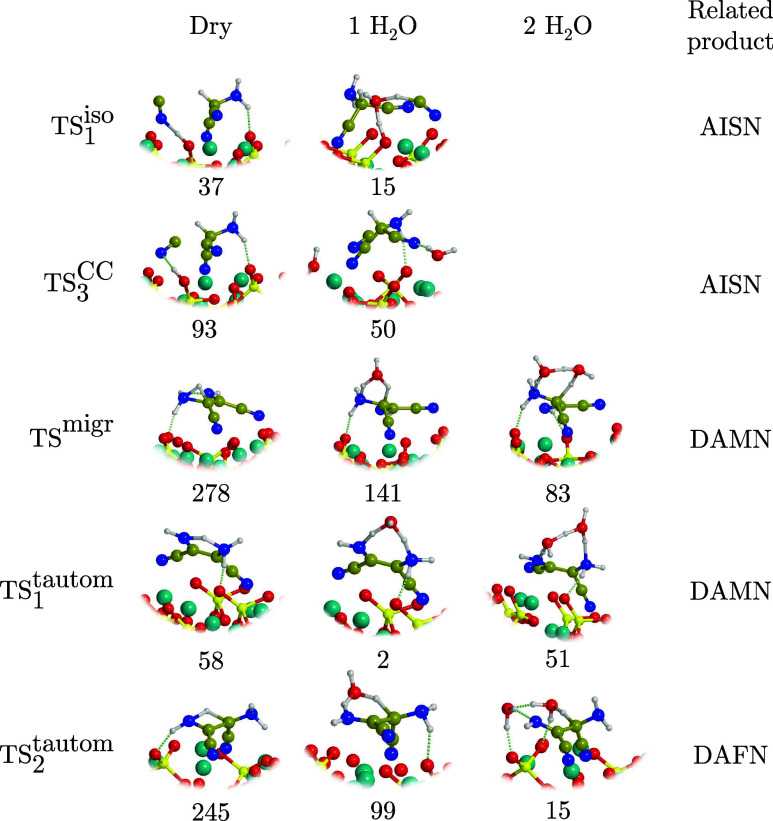
TSs for the conversion of adsorbed HCN and AMN to the two tetrameric forms DAMN and DAFN in dry conditions and when assisted by one and two water molecules. For each structure, the local Δ*G*(300) barrier is reported (in kJ mol^–1^). For the sake of clarity, only the water molecules taking part in the elementary reaction step are depicted. Color coding: dark cyan, Mg atoms; red, O atoms; yellow, Si atoms; blue, N atoms; brown, C atoms; white, H atoms.

The energetics of the water-assisted processes are shown in Figure 5 (blue profile), while the TS structures adopting such water-assisted proton transfers are shown in Figure 6. The presence of water favors energetically more feasible processes, in which low barriers are associated with both the deprotonation of HCN and the C–C nucleophilic attack (Δ*G* ^‡^(300) = 15 and 54 kJ mol^–1^, respectively). Moreover, water further helps the reaction by making the C–C nucleophilic attack and the subsequent reprotonation a concerted step, similar to what was reported in TS_1_^CC^ and TS_2_^CC^ for the formation of IAN and AMN. A similar trend was described by Kikuchi et al.^23^ who reported a 134 kJ mol^–1^ energy barrier for the CN^–^ + HCN → IAN^–^ addition, which lowers to 120 and 109 kJ mol^–1^ when the TS is reprotonated concertedly by ammonium or hydronium cations, respectively, in accordance with their increasing Brønsted acidity. In our case, an even larger reduction in the energy barriers is obtained (−77 kJ mol^–1^) due to the participation of water as a proton shuttle, stabilized by surface-exposed Mg^2+^. The kinetic boost provided by water occurs at the expense of a slight destabilization of AISN (+8 kJ mol^–1^), which could possibly be recovered on more acidic Mg^2+^ sites.

At this point, AISN can tautomerize into two different *cis*–*trans* conformers, giving rise to DAMN and DAFN, respectively, two possible tetramers of HCN (see Figure 2). The mechanistic details of the two isomerizations are discussed in the following sections.

#### DAMN (*cis*-Product) Formation

The Δ*G*(300) profiles for the AISN + HCN → DAMN reaction are shown in Figure 7 including, in addition to the dry surface, the presence of one (‘1 H_2_O’) and two (‘2 H_2_O’) water molecules assisting the proton transfers. The reaction consists of two subsequent proton transfers, for which the total amount of water molecules in the system is two for ‘1 H_2_O’ and four for ‘2 H_2_O’.

**Figure 7:**
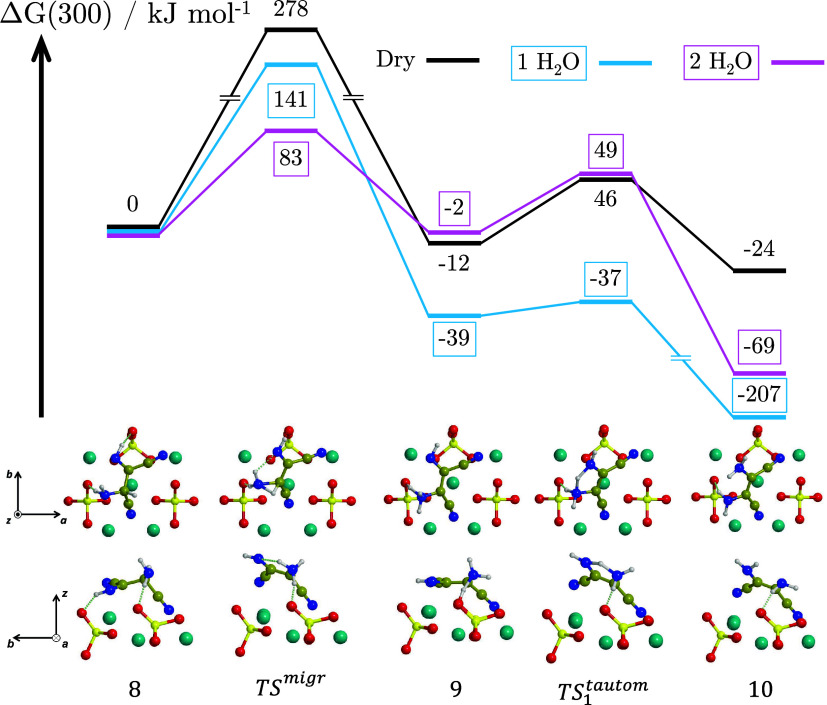
BHLYP-D3­(BJ)//PBEsol-D3­(BJ) Δ*G*(300) profile, in kJ mol^–1^, for the formation of DAMN by tautomerization of AISN adsorbed on the Mg_2_SiO_4_ forsterite (120) surface in dry conditions (‘Dry’, black) and in the presence of one and two water molecules assisting the proton transfers (‘1 H_2_O’, blue; ‘2 H_2_O’, pink). Both the top and lateral views of the dry structures are represented. Relevant structures in the presence of one and two water molecules are shown in Figure 6. For the sake of clarity, only the most exposed surface atoms are shown. Color coding: dark cyan, Mg atoms; red, O atoms; yellow, Si atoms; blue, N atoms; brown, C atoms; white, H atoms.

In the gas-phase, the proton transfer from the C–H group to the NH group is concerted and, accordingly, a high-energy barrier of 242 kJ mol^–1^ has been reported (CBS-QB3//B3LYP/6-31G­(d)).^19^ On forsterite (see Figure 7), without a preliminary rotation around the central C–C bond to bring the C–H closer to the imine group, the tautomerization of AISN from structure **8** of Figure 7 can only proceed through a stepwise process: a proton transfer from the C–H to the vicinal −NH_2_, namely, forming a zwitterion intermediate (structure **9**), followed by a second proton transfer from the so-formed −NH_3_^+^ to NH. Results indicate that the first proton transfer presents an even higher barrier (TS^migr^, Δ*G* ^‡^(300) = 278 kJ mol^–1^) than in the gas-phase due to forming a highly strained 3-member ring.

The second proton transfer, instead, shows a lower energy barrier (TS_1_^tautom^, Δ*G* ^‡^(300) = 46 kJ mol^–1^) as it involves a less strained 5-member ring. Both steps are exoergonic (Δ*G*(300) = −12 and −24 kJ mol^–1^) although less than in the gas-phase (Δ*G*(300) = −40 kJ mol^–1^, see Figure 2), probably due to a greater stabilization of the AISN than DAMN when adsorbed, the latter showing a single N–H···O–SiO_3_ H-bond interaction, while the former two of them.

Due to the fact that the high activation barriers involve strained proton transfers, the possibility that one and two water molecules act as proton transfer assistants was explored (see Figure 7, ‘1 H_2_O’ and ‘2 H_2_O’ profiles, and Figure 6). For the ‘1 H_2_O’-assisted reaction, local barriers were reduced to 141 kJ mol^–1^ and 2 kJ mol^–1^ (TS^migr^ and TS_1_^tautom^, respectively). In the ‘2 H_2_O’-assisted reaction, the first proton transfer energy barrier becomes even lower (83 kJ mol^–1^). In contrast, for the second proton transfer, the barrier is 51 kJ mol^–1^, higher than when assisted by one water. This is due to the geometrical strain imposed by the presence of the second water onto the molecular structure. It has been reported that each water-assisted proton transfer has its own optimum number of waters that best catalyzes the processes, which, among other factors, indeed depends on how well the assistant waters fit into the proton relay chain.^71^ Regarding the thermodynamics, the high exothermicity observed in the presence of water is because, after proton transfer assistance, water molecules tend to adopt more favorable interactions with the product and the surface (Figure S1). This prevents reaching definite conclusions on a preferred stabilization until more exhaustive conformation exploration on the reactants + water and product + water systems will be performed.

#### DAFN (*trans*-Product) Formation

The Δ*G*(300) profile for the formation of DAFN is shown in Figure 8, both on the dry surface and in the presence of one and two water molecules. As only one proton transfer is involved, one water molecule is present in the ‘1 H_2_O’ system and two in the ‘2 H_2_O’ one. The first step for a direct tautomerization from AISN to DAFN is the rotation of the central C–C bond. This requires breaking stable AISN/surface interactions (namely, a H-bond and a CN^–^/Mg^2+^ bond), resulting in an energy barrier of 61 kJ mol^–1^ (TS^rot^) and forming intermediate **11**. This specific step is not reported in the literature,^19^ where the DAMN → DAFN conversion was studied, instead, showing a potential energy barrier as high as 253 kJ mol^–1^ (CBS-QB3//B3LYP/6-31G­(d)), due to rotation around a rigid double bond. The reaction then proceeds with the tautomerization and exothermic formation of DAFN.

**Figure 8:**
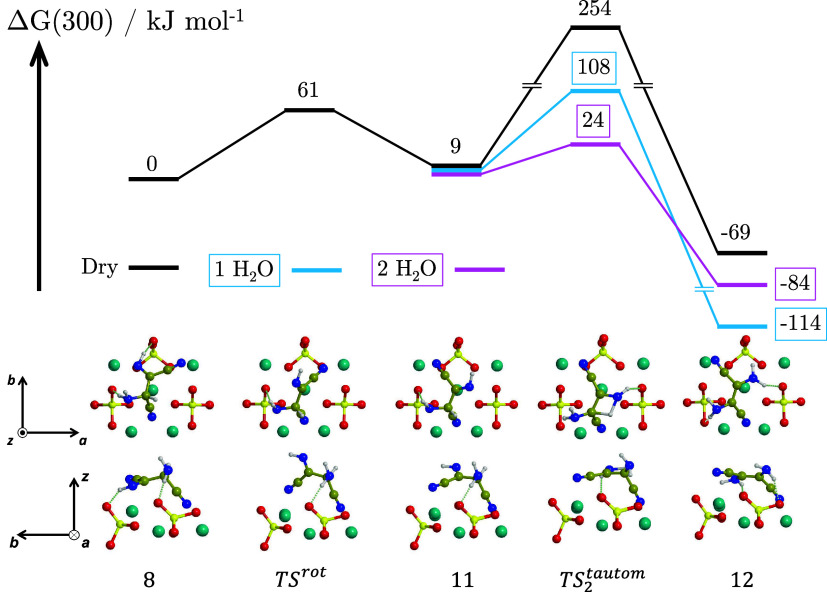
BHLYP-D3­(BJ)//PBEsol-D3­(BJ) Δ*G*(300) profile, in kJ mol^–1^, for the formation of DAFN by tautomerization of AISN adsorbed on the Mg_2_SiO_4_ forsterite (120) surface in dry conditions (‘Dry’, black) and in the presence of one and two water molecules assisting the proton transfers (‘1 H_2_O’, blue; ‘2 H_2_O’, pink). Both the top and lateral views of the dry structures are represented. Relevant structures in the presence of one and two water molecules are shown in Figure 6. For the sake of clarity, only the most exposed surface atoms are shown. Color coding: dark cyan, Mg atoms; red, O atoms; yellow, Si atoms; blue, N atoms; brown, C atoms; white, H atoms.

On the dry surface, the tautomerization presents a high-energy barrier of 254 kJ mol^–1^ due to forming a strained 4-membered ring structure (see TS_2_^tautom^ in Figure 8). Nonetheless, the progressive addition of one and two water molecules assisting the proton transfer reduces the barrier to 108 and 24 kJ mol^–1^, respectively, consistent with a decrease of the strain of the TS structure.

Remarkably, the surface plays a fundamental role in altering the thermodynamics of the process compared with the gas-phase. Indeed, in the latter, DAMN is 4 kJ mol^–1^ more stable than DAFN,^19,70,72,73^ while on the surface, DAFN becomes more stable by 45 kJ mol^–1^, as it finds more anchoring points than DAMN (either H-bonds or CN···Mg).

According to the mechanism of ref 30, to form adenine by DAMN in solution, a DAMN → DAFN isomerization is required first, followed by its cyclization. While a ground-state mechanism in the gas-phase is hindered by a high-energy barrier,^19^ both experiments and theoretical calculations report efficient photocatalytic DAMN → DAFN conversions.^70,72,74,75^ Interestingly, our results indicate that DAFN is easily formed directly from AISN on a microsolvated forsterite surface, preventing the preliminary DAMN → DAFN isomerization.

We would like to stress that the thermodynamics of the HCN oligomerization can differ importantly on other forsterite faces or on other sites of the same surface,^37^ which, in turn, may change the relative stability of the different HCN intermediate oligomers. As the recent experimental measurements^29^ do not allow distinguishing the two isomers (DAMN and DAFN), the hypothesis of a generalized better stabilization of DAFN in spite of DAMN should be checked through a complete and unbiased sampling of the adsorption sites,^76–79^ possibly coupled with a systematic conformational research to account for the many geometrical degrees of freedom of the oligomers.^80–83^ As mentioned above, this applies in particular to the cases of water-assisted mechanisms, in which the thermodynamics is strongly affected by water adsorption on the surface after reaction.

### Kinetic Analysis

The RRKM Arrhenius plots for the most relevant steps of the tetramerization of HCN to DAMN/DAFN between 150 and 350 K are reported in Figure 9. Tunneling effects were also considered, but they are mainly dominant below 150 K (see Figure S2). Accordingly, all the rate constants follow a classical behavior, and all the trends can be correlated with the trends of the energy barriers.

**Figure 9:**
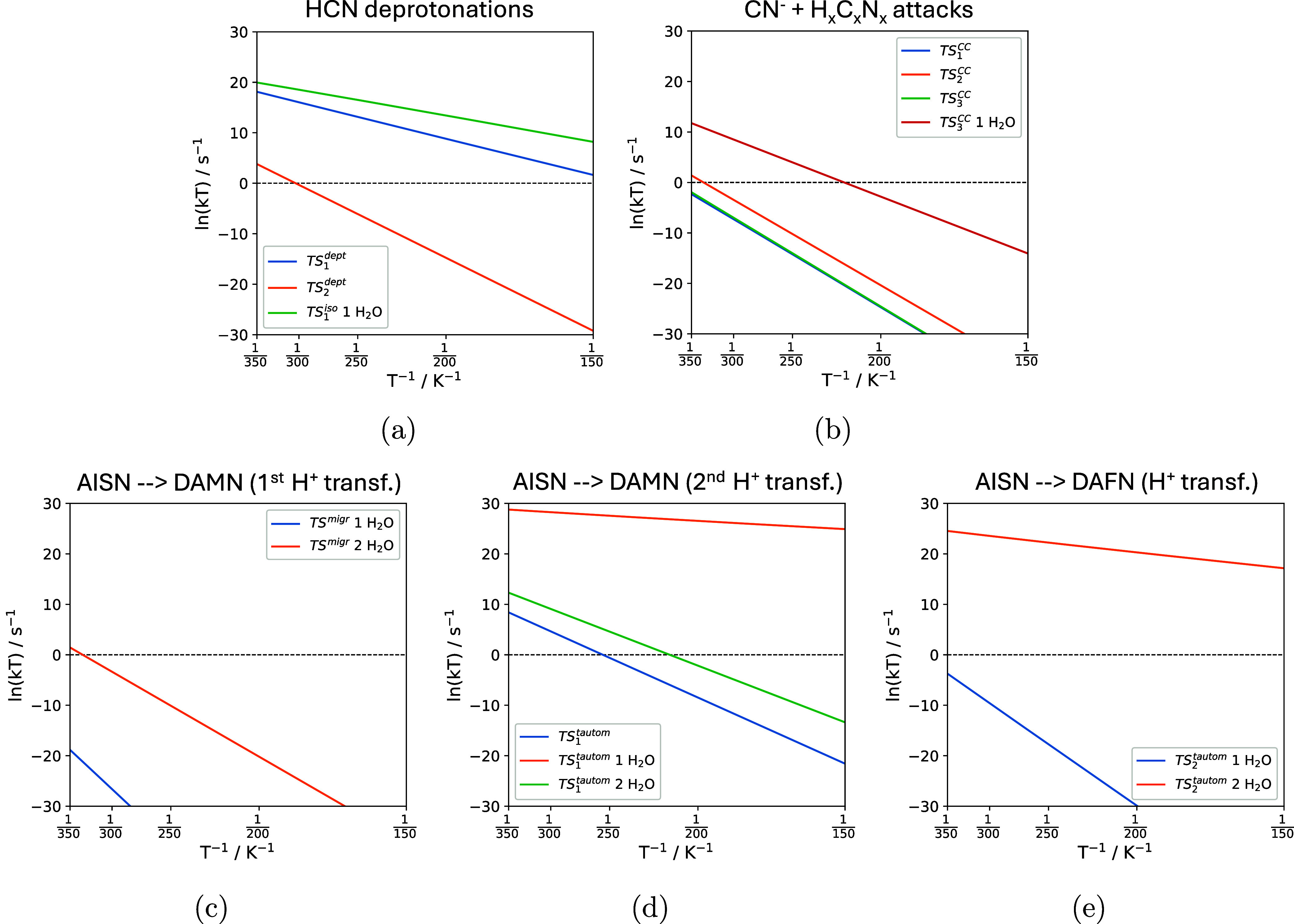
Arrhenius plots for the HCN deprotonations (a), C–C nucleophilic attacks (b), and the intramolecular proton transfers TS^migr^ (c), TS_1_^tautom^ (d), and TS_2_^tautom^ (e). When present, the plots for water-assisted proton transfer are also represented.

The different rates associated with the HCN deprotonation steps (TS_1_^dept^, TS_2_^dept^, and TS_1_^iso^ ‘1 H_2_O’) can be rationalized by the propensity of the proton acceptors. Indeed, the lowest rate constants are computed for TS_2_^dept^, where the proton is transferred to the = NH group of the IAN, while larger rate constants are obtained for TS_1_^dept^, in which the proton acceptor is a surface-exposed basic O^2–^ atom. The largest rate constants are obtained when the proton transfer to the surface is assisted by one water molecule (TS_1_^iso^ ‘1 H_2_O’), consistent with the reduction of the energy barriers.

As previously commented, all nucleophilic attacks forming C–C bonds in dry surface conditions show similar barriers (between 78 and 93 kJ mol^–1^). Accordingly, the corresponding Arrhenius plots are also similar (see TS_1_^CC^, TS_2_^CC^, and TS_3_^CC^ plots). In the latter, the presence of water (TS_3_^CC^ ‘1 H_2_O’) allows for a more favorable process and hence the faster kinetics compared to dry surface conditions.

The kinetics for TS^migr^, TS_1_^tautom^, and TS_2_^tautom^ further highlights the extracatalytic role of water, as almost none of the reactions would take place at appreciable velocity if the proper number of water molecules helping the reaction is not reached (speed up of 20 to 30 orders of magnitude).

Based on this kinetic analysis, it can be stated that the catalytic activity of the forsterite surface, aided by water molecules, toward the oligomerization of HCN is effective above 300 K, corresponding to kinetic constants close to or higher than 1 s^–1^. Indeed, at these temperatures, the basic/acidic behavior of the surface helps the subsequent activation/addition of the HCN monomer to the H_*x*_C_*x*_N_*x*_ neutral oligomers with favorable kinetics. Thus, the thermodynamically stable DAMN and DAFN products can only be synthesized at relatively high temperatures and in the presence of water molecules. Remarkably, such conditions are easily encountered in evolved astronomical environments where possible HCN products have been detected (see Introduction). Indeed, a qualitative Eyring kinetic comparison at 300 K, based on potential energies computed here and, in the gas-phase, by Jung and Choe,^19^ shows accelerations of around ∼20–40 orders of magnitude for the rate-determining steps of the reaction (see Table S2). Accordingly, the mechanisms reported here can be considered reliable models, explaining the extraterrestrial, mineral-catalyzed HCN prebiotic tetramerization.

## Conclusions

In the present work, the mechanistic details, including energetics, of the prebiotic oligomerization of HCN to its stable tetramers DAMN and DAFN catalyzed by the Mg_2_SiO_4_ forsterite (120) surface are reported for the first time. The reaction is effectively triggered when HCN deprotonates upon adsorption by the action of surface-exposed basic O^2–^ atoms, forming surface SiOH^+^···CN^–^ H-bonded ion pairs. The growth of the molecule is facilitated by incorporating CN^–^ in the H_*x*_C_*x*_N_*x*_ neutral oligomers through nucleophilic C–C attacks, helped by surface-exposed acidic Mg^2+^ centers as well as the presence of individual water molecules assisting proton transfer processes, stabilizing the TSs. RRKM analysis shows that the resulting reactions require temperatures above 300 K to occur with appreciable rate constants (*k* ≥ 1 s^–1^), in accordance with experimental observations. Optimal conditions in terms of temperature and availability of water-assistant proton transfer molecules can be found on warm and evolved rocky bodies such as asteroids, comets, and planets.

## Supplementary Material



## Data Availability

The chemical processes simulated here thus support a reliable reaction model to explain the catalytic tetramerization of HCN up to DAMN and DAFN, which, in turn, is possibly related to the presence of nucleobases and other biomolecules in warm rocky environments. A Zenodo repository with all the structures discussed in this work is provided (DOI: 10.5281/zenodo.15624562).

## Notes

Test on the performances of the BHLYP-D3­(BJ) scheme, structures for the AISN → DAMN conversion on the surface when proton transfers are aided by one water molecule, comparisons between the Arrhenius plots between 150 and 350 K with and without Tunnelling correction, barriers and kinetic constants for the rate-determining step of each reaction when in the gas-phase and on the surface and reaction potential energy, enthalpy at 0 K and Gibbs energy at 300 K when in the gas-phase and on the surface. (PDF)
