# Supplementary material for: Cosmic Silicate Surfaces Catalizing Prebiotic Reactions: Atomistic Modeling on the Polymerization of HCN
Source: ACS Earth Space Chem. 2025 Nov 8;9(11):2567–78. doi: 10.1021/acsearthspacechem.5c00166 (PMC12641543; doi:10.1021/acsearthspacechem.5c00166)
Supplement: Supplementary file 1 [file sp5c00166_si_001.pdf]

Cosmic silicate surfaces catalising prebiotic reactions: atomistic modeling on the polymerization of HCN

Supporting Information

Niccolò Bancone [†,‡] Stefano Pantaleone [‡] Gerard Pareras [†] Piero Ugliengo [‡] Albert Rimola [*,†] and Marta Corno [*,‡]

[†] *Departament de Química, Universitat Autònoma de Barcelona, 08193, Bellaterra, Catalonia, Spain*

[‡] *Dipartimento di Chimica and Nanostructured Interfaces and Surfaces (NIS) Centre, Università degli Studi di Torino, via P. Giuria 7, 10125, Torino, Italy.*

E-mail: albert.rimola@uab.cat; marta.corno@unito.it

Phone: +34-935813723; +39-0116702439

Table S1: Relative potential energies (ΔE), in kJ mol^{-1}, of reactants (R), transitions states (TS) and products (P) of the rate-determining steps for the HCN tetramerization, computed at the PBEsol-D3, BHLYP-D3 and DLPNO-CCSD(T) level of theories. The first reaction is the CN^- + HCN→ IAN^- nucleophilic attack and the second reaction is the AISN→ DAFN isomerisation through intramolecular proton transfer, both modeled on a small forsterite nanocluster. PBEsol-D3 data were obtained with the CP2K code (v. 9.1.0), adopting a MOLOPT-DZVP basis set for surface atoms (Mg, Si and O) and MOLOPT-TZVP for adsorbates (H, C and N). BHLYP-D3 data were obtained with the CRYSTAL23 code, adopting the Ahlrichs VTZP for adsorbates atoms and a Pople basis set with polarization functions for the surface. DLPNO-CCSD(T) data were obtained with the ORCA6.0 code, adopting the aug-cc-pVTZ basis set.

		ΔE (kJ mol^{-1})		
		PBEsol-D3	BHLYP-D3	DLPNO-CCSD(T)
	R	0.0	0.0	0.0
CN^- + HCN→ IAN^-	TS	1.3	24.8	24.0
	P	-31.2	11.0	16.0
	R	0.0	0.0	0.0
AISN→ DAFN	TS	200.9	269.8	265.5
	P	-86.8	-52.8	-24.4

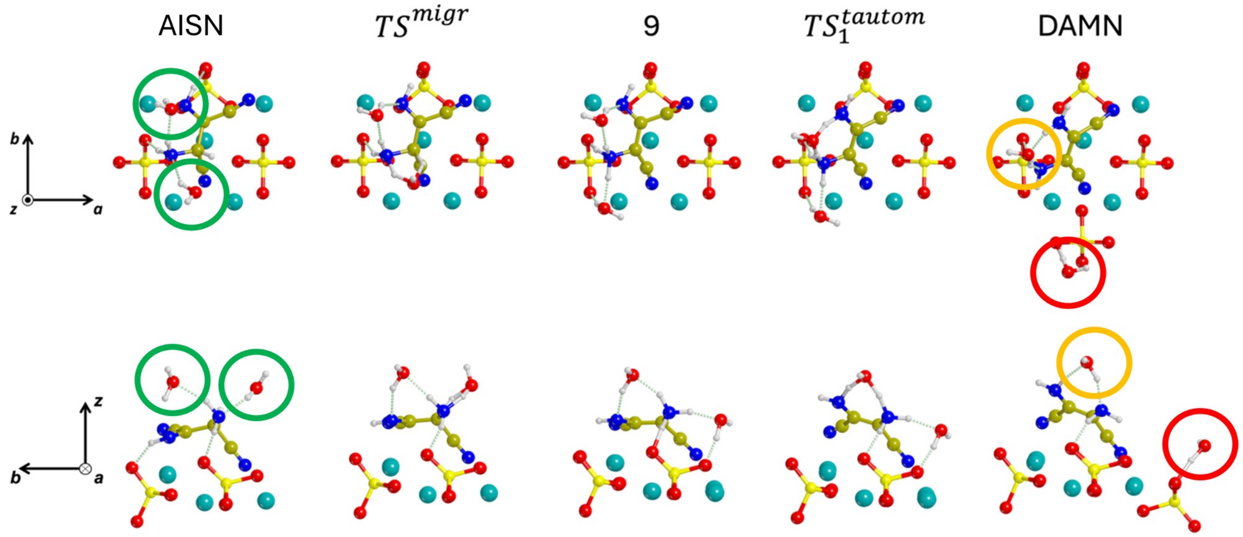

Figure S1: Top and side views of the structures involved in the conversion of AISN to DAMN when each intramolecular proton transfer is assisted by one water molecule. Water molecules are loosely bound to the reactants when placed in the optimal configuration to assist the transfers (green circles). At the product geometry, one water molecule assumes a double H-bond interaction with DAMN (yellow circle) while the other becomes strongly adsorbed on the surface (red circle), affecting relevantly the thermodynamic of the reaction.

Table S2: Potential energy barriers (ΔE, in kJ mol^{-1}) for the rate-determining steps at each stage of the oligomerization of HCN (see Figure 2 in the main text). Values in the gas-phase at the CBS-QB3//B3LYP/6-31G(d) level of theory are taken from ref. 1, 'Dry', '1 H_2O' and '2 H_2O' are referred to the reactions on the (120) forsterite surface studied in this work at the BHLYP-D3//PBEsol-D3 level. Kinetic constants at 300 K (k(300), in s^{-1}), are computed on the electronic energies according to the Eyring equation.

Reaction step	Gas [1]		Dry		1 H_2O		2 H_2O	
	ΔE	k(300)	ΔE	k(300)	ΔE	k(300)	ΔE	k(300)
$2\,HCN \longrightarrow IAN$	312	3.0×10^{-42}	90	1.3×10^{-3}	-	-	-	-
$HCN + IAN \longrightarrow AMN$	202	4.2×10^{-23}	82	3.3×10^{-2}	-	-	-	-
$HCN + AMN \longrightarrow AISN$	269	9.1×10^{-35}	88	3.0×10^{-3}	55	1.7×10^{3}	-	-
$AISN \longrightarrow DAMN$	242	4.6×10^{-30}	293	6.0×10^{-39}	153	1.4×10^{-14}	106	2.2×10^{-6}
$AISN \longrightarrow DAFN$	253	5.5×10^{-32}	249	2.8×10^{-31}	122	3.6×10^{-9}	29	5.6×10^{7}

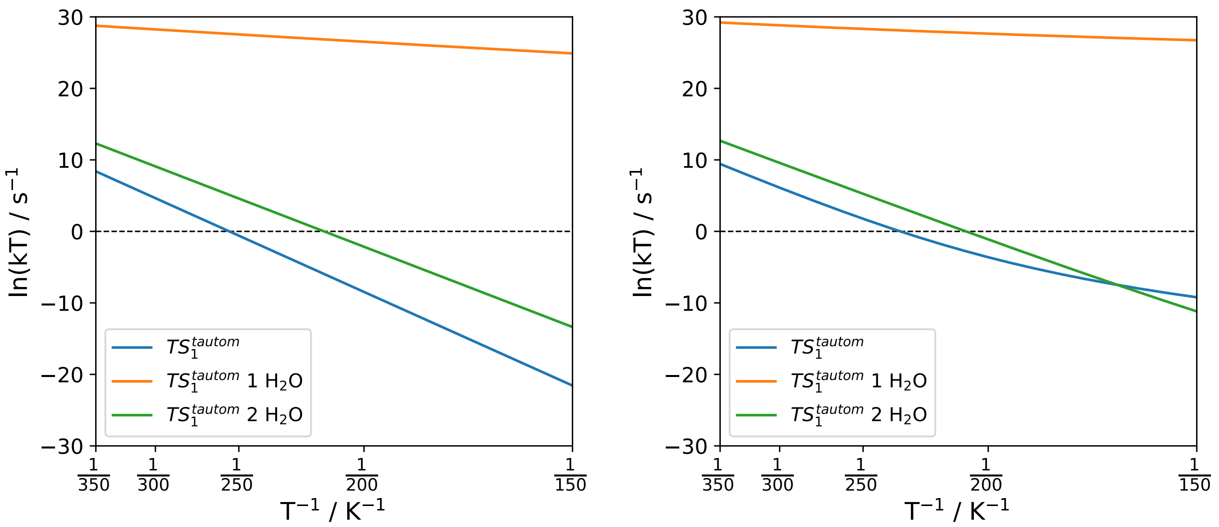

Figure S2: Arrhenius plots for the the steps associated to TS_1^{tautom} in dry conditions (TS_1^{tautom}) and when internal proton transfers are aided by one (TS_1^{tautom}, '1 H_2O') or two (TS_1^{tautom}, '2 H_2O') water molecules. Left: plot computed classically. Right: plot corrected for the Eckart tunnelling[2,3].

Table S3: Potential reaction energies (ΔE), reaction enthalpies at 0 K (ΔH(0)) and Gibbs reaction energies at 300 K (ΔG(300)) in kJ mol^{-1} for each elementary step of the HCN oligomerization to DAMN and DAFN. For each reaction, the values computed in the gas-phase (see Figure 2 in the main text) are compared with those on the surface (Figures 3-5,7-9).

Reaction step	$2\,HCN \longrightarrow IAN$					
Phase	Gas		Surface			
Structure	HCN···HCN	IAN	**1**	**2**	**3**	
ΔE	0	-24	0	-46	-40	
ΔH(0)	0	-10	0	-45	-27	
ΔG(300)	0	-2	0	-45	-21	
Reaction step	$HCN + IAN \longrightarrow AMN$					
Phase	Gas		Surface			
Structure	HCN···IAN	AMN	**4**	**5**	**6**	
ΔE	0	-72	0	36	-27	
ΔH(0)	0	-61	0	31	-14	
ΔG(300)	0	-53	0	33	-11	
Reaction step	$HCN + AMN \longrightarrow AISN$					
Phase	Gas		Surface			
Structure	HCN···AMN	AISN	**7**	7^{iso}	8^{iso}	**8**
ΔE	0	-42	0	41	5	-9
ΔH(0)	0	-31	0	39	8	1
ΔG(300)	0	-26	0	38	13	7
Reaction step	$AISN \longrightarrow DAMN$					
Phase	Gas		Surface			
Structure	AISN	DAMN	**8**	**9**	**10**	
ΔE	0	-42	0	-10	-28	
ΔH(0)	0	-41	0	-11	-25	
ΔG(300)	0	-40	0	-12	-24	
Reaction step	$AISN \longrightarrow DAFN$					
Phase	Gas		Surface			
Structure	AISN	DAFN	**8**	**11**	**12**	
ΔE	0	-39	0	7	-73	
ΔH(0)	0	-38	0	8	-71	
ΔG(300)	0	-36	0	9	-69	

References

(1) Jung, S. H.; Choe, J. C. Mechanisms of prebiotic adenine synthesis from HCN by oligomerization in the gas phase. *Astrobiology* **2013**, *13*, 465–475.

(2) Eckart, C. The penetration of a potential barrier by electrons. *Phys. Rev.* **1930**, *35*, 1303.

(3) Johnston, H. S.; Heicklen, J. Tunnelling corrections for unsymmetrical Eckart potential energy barriers. *J. Phys. Chem.* **1962**, *66*, 532–533.
